# Remyelination Improves Voiding Dysfunction in a Mouse Model of Multiple Sclerosis

**DOI:** 10.1093/geroni/igab046.2419

**Published:** 2021-12-17

**Authors:** Ramalakshmi Ramasamy, Cara Hardy, Stephen Crocker, Phillip Smith

**Affiliations:** UConn Health, Farmington, Connecticut, United States

## Abstract

Multiple sclerosis (MS) is a chronic inflammatory demyelinating disease of the central nervous system (CNS). Of note, over 80% of MS patients have urinary symptoms as one of their earliest symptoms. Since MS patients often live into older age, urinary incontinence and retention are significant problems affecting their quality of life. Although several studies show that inflammatory-demyelinating animal models of MS develop bladder dysfunction, the confounding influence of systemic inflammation in these models limits potential interpretation on the contribution of CNS-myelination to bladder dysfunction. We sought to address this knowledge gap using the cuprizone model of demyelination and remyelination. C57Bl/6 mice were treated with dietary cuprizone (0.2%w/w) for four weeks to induce demyelination. One group was allowed four additional weeks for recovery and remyelination. We performed voiding spot assay (VSA), urethane-anesthetized cystometry, and CNS-histology to assess demyelination-induced differences in urinary performance. We observed that cortical demyelination causes significant aberrance in voiding behavior (conscious cortical control) characterized by increased micturition frequency and reduced volume per micturition. Interestingly, remyelination restored healthy bladder function. However, there were no significant changes in the cystometric parameters (brainstem reflex) between the treatment groups. While MS is not classically considered a disease of aging, extending the longevity of these patients has not been reciprocated with improved treatments for their most-bothersome conditions, notably urinary symptoms that persist throughout life. Our data represent a novel compelling connection and strong correlation between CNS-myelination and cortical control of bladder function, which has potential implications in MS, aging, and aging-associated neurological disorders.

